# Successful right transbrachial cerebral angiography in a patient with aberrant right subclavian artery: A technical note

**DOI:** 10.1016/j.radcr.2025.02.008

**Published:** 2025-03-11

**Authors:** Naomichi Tamura, Toru Umehara, Yoshihiro Yano, Toshiaki Fujita, Haruhiko Kishima

**Affiliations:** a Department of Neurosurgery, Hanwa Memorial Hospital, Osaka, Japan; b Department of Neurosurgery, Osaka University Graduate School of Medicine, Suita, Osaka, Japan; c Department of Neurosurgery, Osaka International Cancer Institute, Osaka, Japan

**Keywords:** Aberrant right subclavian artery, Transbrachial cerebral angiography, Technical difficulties, Concomitant vascular anomaly, Arteriovenous malformation

## Abstract

Aberrant right subclavian artery (RSCA) is a congenital anomaly in which the RSCA arises as the final branch of the aortic arch, the presence of which makes cerebral angiography (CAG) via the right transbrachial approach (RTBA) difficult to perform. Herein, we present a case of aberrant RSCA in which the right transbrachial CAG was successfully executed and review its technical details in consideration of the anatomy of the aberrant RSCA. A 51-year-old man was admitted to our hospital with an unexplained subcortical hemorrhage in the left parietal lobe. Diagnostic CAG was performed via the RTBA, leading to the diagnosis of aberrant RSCA. Despite some technical difficulty, the Simmons curve was formed at an acute angle between the aberrant RSCA and the aortic arch. Once the Simmons curve is formed, torque control of the catheter can result in the bilateral common carotid artery (CCA) cannulation without switching to a transfemoral approach. Left CCA angiography confirmed an arteriovenous fistula in the distal left anterior cerebral artery. Subsequently, the suspected source of intracerebral bleeding and associated hematoma were surgically resected. Early detection of aberrant RSCA is crucial when using the RTBA. Depending on the procedure of catheterization, the right transbrachial CAG can be performed even in patients with aberrant RSCA; therefore, its continued usage seems worthy of consideration after setting a time limit.

## Introduction

Aberrant right subclavian artery (RSCA) is a congenital anomaly occurring in approximately 0.5%–1% of the population, in which the RSCA arises as the final branch of the aortic arch [1,2]. This anomaly is more common in fetuses with chromosomal defects, particularly those with trisomy 21 [3]. Embryologically, the normal RSA forms from the right brachiocephalic artery after the right dorsal aorta regresses distally to the seventh right intersegmental artery. An aberrant RSCA develops when the seventh right intersegmental artery and the dorsal aorta caudal to it remain as the RSCA due to the regression of the right fourth aortic arch [4]. Generally, patients with an aberrant RSCA do not present with any apparent clinical symptoms; however, in some cases, Kommerell's diverticulum, located at the origin of the aberrant RSCA, can compress the esophagus or trachea, causing difficulties in swallowing or breathing [5].

The presence of an aberrant RSCA typically makes it difficult to perform cerebral angiography (CAG) and other therapeutic interventions via the right transbrachial approach (RTBA) or the transradial approach, mainly because it crosses the midline with an abnormally long and tortuous course [6]. Few studies have focused on technical tips when encountering an unexpected aberrant RSCA during the RTBA, and discontinuing the procedure or switching to a transfemoral approach (TFA) have been reasonable alternatives. Herein, we present a case where the right transbrachial CAG for aberrant RSCA was successfully performed and review its technical details in consideration of the anatomy of the aberrant RSCA.

## Case presentation

**Fig. 1:**
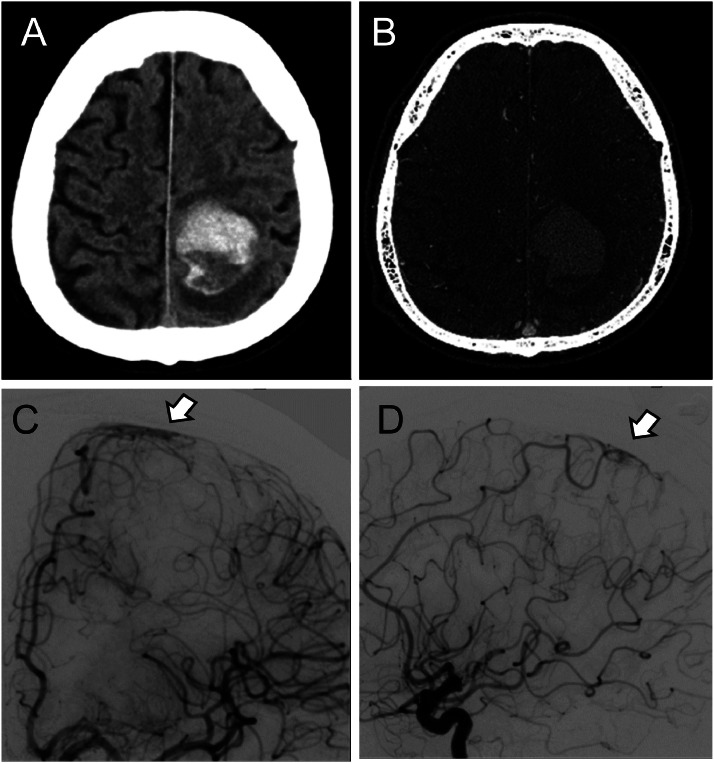
The images above (A, B) are axial CT scans of the head obtained upon admission. (A) CT demonstrating a subcortical hemorrhage (40 mm) in the left parietal lobe. (B) CTA showing no vascular abnormalities identified as the source of bleeding. The images below (C, D) are preoperative DSA. The left ICA angiograms in the anteroposterior (C) and lateral (D) projections reveal a fistula at the distal left anterior cerebral artery (arrowheads), suggesting a source of bleeding.

**Fig. 2:**
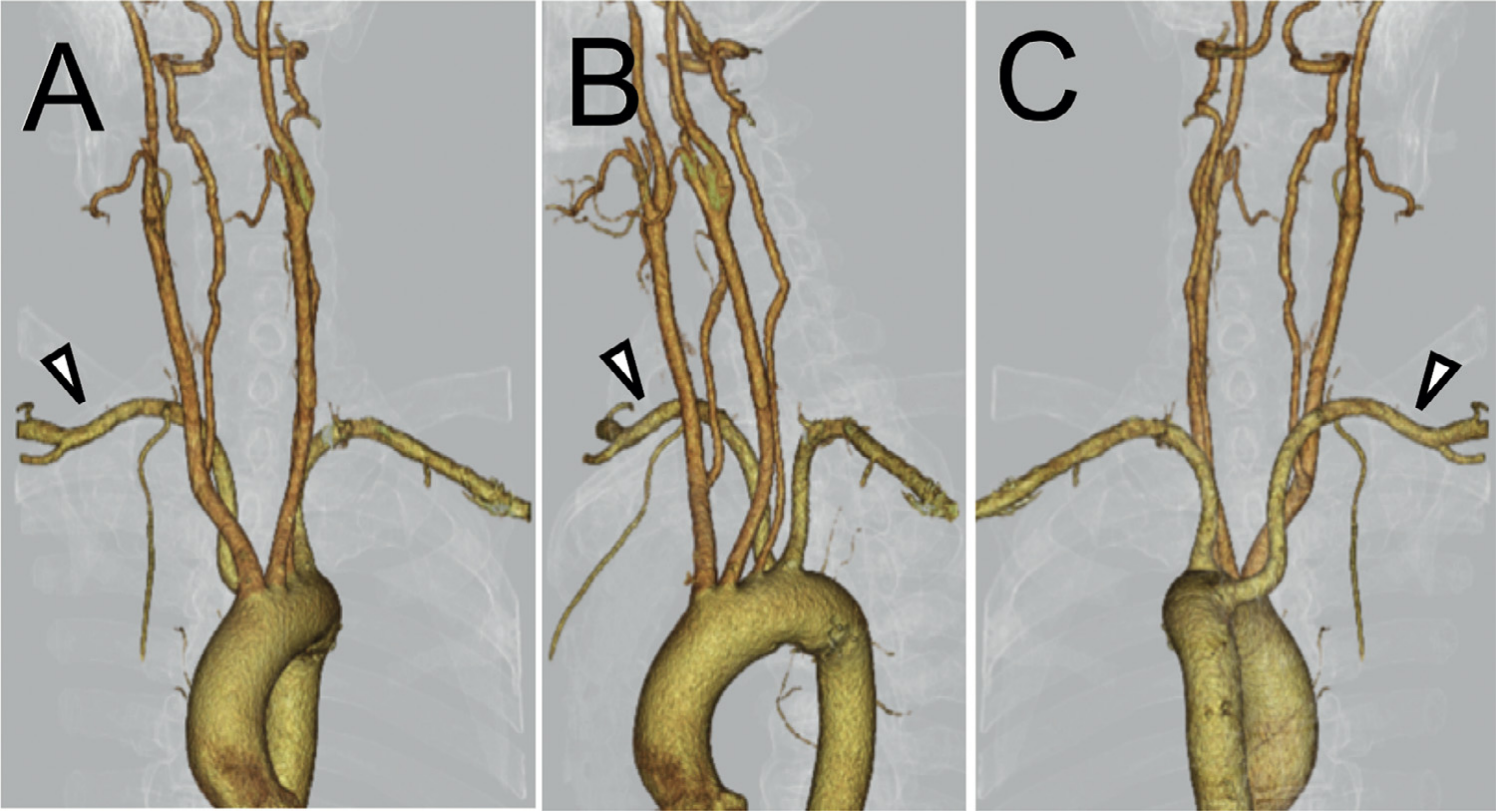
3D-CTA of the chest in anteroposterior (A), left lateral (B), and posteroanterior (C) projection confirms the presence of an aberrant RSCA (arrowhead). The aberrant RSCA arises from the descending aorta in the superoposterior direction.

A 51-year-old man with a history of diabetes mellitus but no history of hypertension was admitted to our hospital with sudden onset right-sided hemiparesis. Computed tomography (CT) of the head revealed a subcortical hemorrhage in the left parietal lobe without a suspected source for the bleeding on CT angiography (CTA). We performed a diagnostic CAG via the brachial artery on the fourth day of admission. Despite encountering an aberrant RSCA, CAG was successfully performed purely via the RTBA, confirming an arteriovenous shunt at the distal left anterior cerebral artery as the intracerebral bleeding source (Fig. 1). CTA of the chest subsequently confirmed the presence of the aberrant RSCA (Fig. 2). Technical details of the right transbrachial CAG for aberrant RSCA are described in the following section.

On the tenth day of his hospital stay, the patient underwent surgical resection of the arteriovenous shunt. A pathological examination was subsequently performed, and the results were consistent with an arteriovenous malformation (AVM). On the 20th day of his hospital stay, the patient underwent follow-up angiography via the TFA, confirming the absence of the AVM. On the 62nd day of his hospital stay, the patient was discharged home following inpatient rehabilitation, with a modified Rankin Scale score of 1.

## Right transbrachial cerebral angiography procedure for aberrant RSCA

A 4-Fr short sheath (Zeon Medical, Tokyo, Japan) was inserted into the right brachial artery, and a Simmons-type 4-Fr catheter (SY3; Goodman, Aichi, Japan) with a 0.035-inch, 150-cm angle-type guidewire (Radifocus Guidewire M standard type; Terumo Corporation, Tokyo, Japan) was placed into the RSA. The guidewire was exclusively inserted into the descending aorta; therefore, the Simmons catheter was withdrawn and a 4-Fr pigtail catheter (Goodman) was advanced to the origin of the RSCA. Aortography using the pigtail catheter revealed that the RSCA was the final branch of the aortic arch, suggestive of an aberrant RSCA. After manually forming the tip of the guidewire into a J-shape, the guidewire could be induced into the ascending aorta and turned over at the aortic valve to form a Simmonds catheter shape; however, the Simmons catheter repeatedly herniated into the descending aorta owing to the tortuosity at the origin of the aberrant RSCA. Accordingly, we slowly retracted and twisted the Simmons catheter instead, which allowed us to successfully access the right common carotid artery (CCA).

**Fig. 3:**
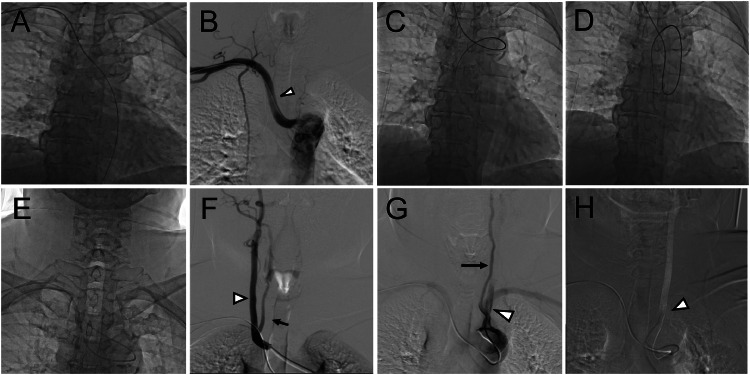
The radiographic imaging above (A–D) was obtained before accessing the carotid arteries. (A) X-ray image showing that the guidewire was exclusively advanced into the descending aorta. (B) Aortography revealing that the RSCA (arrowhead) is the final branch of the aortic arch. (C) X-ray image showing that the guidewire successfully advanced into the ascending aorta. (D) X-ray image subsequently shows the guidewire and catheter intolerantly herniating into the descending aorta. The radiographic imaging below (E–H) was obtained after selecting carotid arteries. (E) X-ray image showing that the catheter selects the right CCA while forming a Simmons curve. (F) Right CCA angiography showing a common trunk shared by the right CCA (arrowhead) and the right VA (black arrow). (G) Intra-aortic injection just distal to the origin of the right CCA showing, in order, the left CCA (arrowhead), the left VA originating directly from the aortic arch (black arrow), and the left subclavian artery. (H) DSA overlay showing that the right CCA is successfully selected by the catheter (arrowhead).

**Fig. 4:**
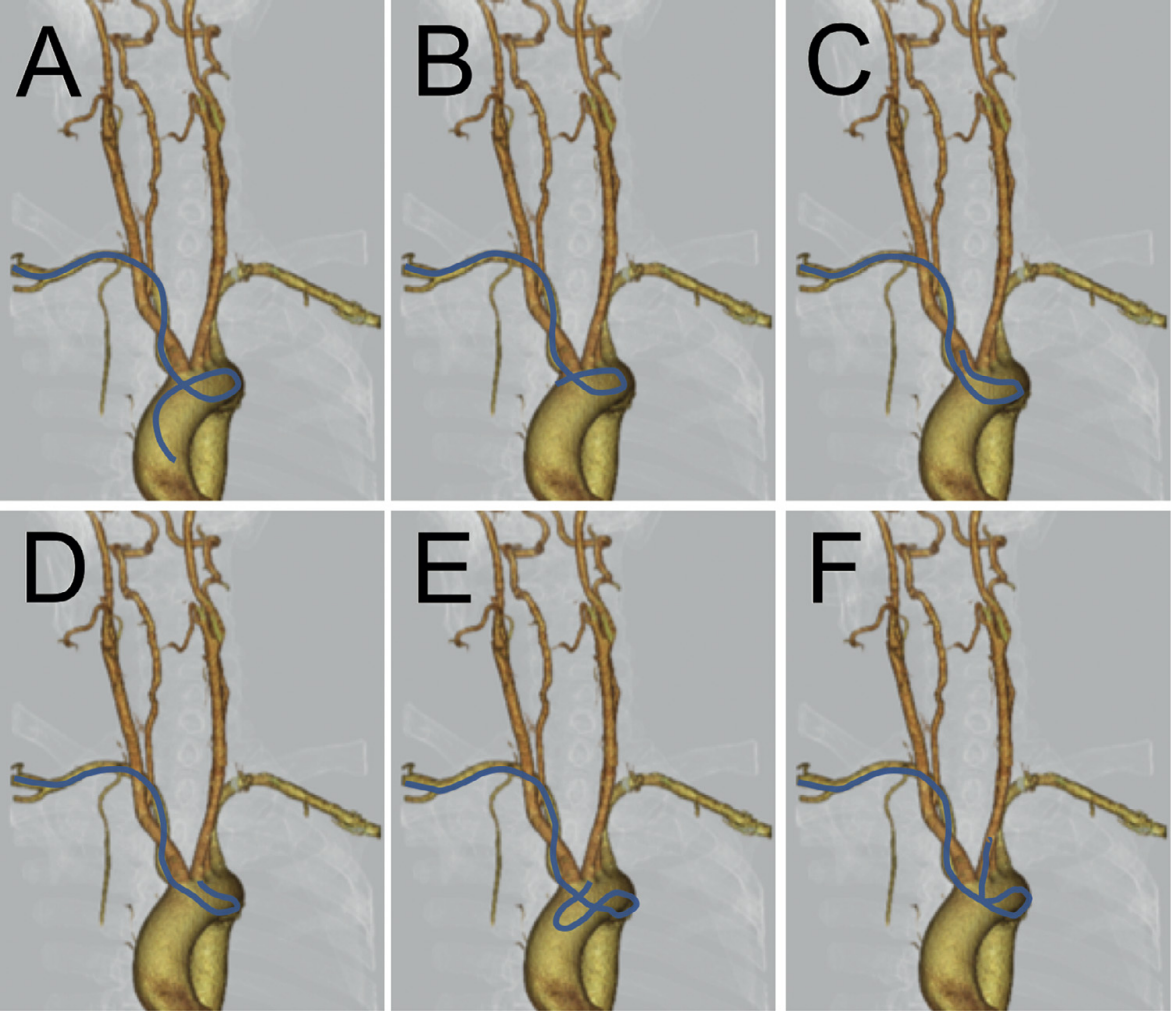
Schematic illustration of catheterization to access carotid arteries. The summarized behavior of the Simmons catheter was overlain on the 3D CTA image of the chest in an anteroposterior projection. (A, B) After the catheter was slowly retracted from the ascending aorta, the Simmons curve was formed at an acute angle between the aberrant RSCA and the aortic arch. (C) Once the Simmons curve is formed, torque control of the catheter can result in right CCA cannulation. (D) The catheter is pushed out of the right CCA and dropped into the aortic arch while maintaining the Simmons curve. (E) By torqueing and advancing the catheter, the Simmons curve is guided into the ascending aorta, utilizing the outer wall of the descending aorta as a fulcrum. (F) The left CCA was accessed with a catheter using guidewire-assisted induction.

Angiography of the right CCA revealed no abnormalities in the intracranial vessels. However, a common trunk shared by the right CCA and vertebral artery (VA) was observed. Subsequent angiography of the aortic arch confirmed the positional relationship between the left CCA, the left VA originating directly from the aortic arch, and the left subclavian artery. After the Simmons curve was dropped into the descending aorta, the left CCA was successfully accessed by twisting and advancing the catheter. Strong support from the outer wall of the descending aorta enabled further advancement of the guidewire into the left ICA, while a twisting motion resolved the distortion of the catheter to allow for successful advancement into the ICA. The angiograms before and after selecting carotid arteries are respectively shown in Figure 3, and the schematic illustration of catheterization is also shown, using the chest 3D CTA image in an anteroposterior projection, as shown in Figure 4.

## Discussion

In practice, a tortuous RSCA related to arteriosclerosis is a frequent limiting factor of guidewire advancement into the ascending aorta during the CAG via the RSCA. Thus, it would be worthwhile to attempt some maneuvers, such as deep breathing or leftward head rotation, which are reported to be clinically effective [7,8]. If the ascending aorta still cannot be accessed, aortography should be performed using a pigtail catheter for early detection of aberrant RSCA. The aortic arch and aberrant RSCA generally form an acute angle, and a standard-angled guidewire is of limited use for accessing the ascending aorta. Therefore, manually bending its distal tip can be effective. Alternatively, a steerable guidewire may be more useful. Even after the ascending aorta is successfully accessed, a subsequent challenge is the lack of reachability into the aortic valve. In our case, the catheter repeatedly herniated into the descending aorta when trying to conventionally form the Simmons curve at the aortic valve. Accordingly, we formed the Simmons curve by utilizing the acute angle at the origin of the aberrant RSCA and were able to access the right CCA. Once the Simmons curve was formed, it was then possible to select carotid arteries by push-pull and torque control of the catheter as shown in Figure 4.

There are three courses that the aberrant RSCA can follow after branching from the aortic arch: posterior to the esophagus (80%), between the esophagus and trachea (15%), and anterior to the trachea (5%) [9]. Additionally, the aberrant RSCA diverges from the aortic arch in either a superoposterior (88%) or inferoposterior (12%) direction against the aortic arch [10]. In our case, the aberrant RSCA follows the most common course, posterior to the esophagus in the superoposterior direction (Fig. 3). No reports describe whether these variations in the aberrant RSCA course affect the catheterization procedure. The course which runs anterior to the trachea or with inferoposterior divergence may lead to a more acute angle between the ARSA and the aortic arch, making it more challenging to navigate the guidewire to the ascending aorta.

In light of arterial anomalies concomitant with aberrant RSCA, our case showed a relatively infrequent pattern, in that the right CCA and VA shared a common trunk and the left VA originated directly from the aorta [11]. Tsai et al. reported that 15.7% of patients with aberrant RSCA exhibited anomalies in the origins of the VA, while only 3.9% of these were bilateral [12]. Prior knowledge of the frequency of potential concomitant arterial anomalies with the aberrant RSCA may help save procedural time during catheterization, potentially lowering complication rates. To the best of our knowledge, there have been no reported cases of aberrant RSCA in conjunction with AVMs other than our case. Nevertheless, we concluded that this was a chance finding and, therefore, did not conduct genetic testing, owing to the absence of other distinctive congenital conditions and a negative family history suggesting minor implications for genetic disorders.

As described above, the right transbrachial CAG can be performed even in patients with an aberrant RSCA. However, catheterization via the aberrant RSCA ordinarily prolongs procedural time and runs the risk of iatrogenic complications such as vascular dissection and intramural hematoma [6,13]. Abandoning the RTBA and/or switching to the TFA should, therefore, be judiciously considered. Procedure times longer than 60 min have been reported to be associated with a risk of neurologic events following CAG [14]. Thus, it is a reasonable suggestion to consider discontinuation when the time from puncture to the time of accessing the target vessels takes longer than 30 min due to the presence of an aberrant RSCA.

In summary, early detection of an aberrant RSCA is crucial during the CAG via the RTBA. Despite some technical difficulties, the right transbrachial CAG has the potential to be performed even in patients with an aberrant RSCA, and the continued use of RTBA seems to be worthy of consideration after setting a time limit.

## Patient consent

Written informed consent was obtained from the patient for the publication of this case and any relevant images.

## References

[1] Asherson N. (1979). David Bayford. His syndrome and sign of dysphagia lusoria. Ann R Coll Surg Engl.

[2] Rowe D.M., Becker G.J., Scott J.A., Conces D.J. (1988). Right subclavian steal associated with aberrant right subclavian artery. AJNR Am J Neuroradiol.

[3] Scala C., Leone Roberti Maggiore U., Candiani M., Venturini P.L., Ferrero S., Greco T. (2015). Aberrant right subclavian artery in fetuses with Down syndrome: a systematic review and meta-analysis. Ultrasound Obstet Gynecol.

[4] Bae S.B., Kang E.J., Choo K.S., Lee J., Kim S.H., Lim K.J. (2022). Aortic arch variants and anomalies: embryology, imaging findings, and clinical considerations. J Cardiovasc Imaging.

[5] Polguj M., Chrzanowski L., Kasprzak J.D., Stefanczyk L., Topol M., Majos A. (2014). The aberrant right subclavian artery (arteria lusoria): the morphological and clinical aspects of one of the most important variations–a systematic study of 141 reports. ScientificWorldJournal.

[6] Kassimis G., Sabharwal N., Patel N., Banning A. (2013). Aberrant right subclavian artery hematoma following radial catheterization. JACC Cardiovasc Interv.

[7] Parikh D.S., Gandhi K., Shroff A. (2019). Radial percutaneous coronary intervention in complex arm and chest vasculature: tips and tricks. Curr Treat Options Cardiovasc Med.

[8] Brunet M.C., Chen S.H., Peterson E.C. (2020). Transradial access for neurointerventions: management of access challenges and complications. J Neurointerv Surg.

[9] Stone W.M., Brewster D.C., Moncure A.C., Franklin D.P., Cambria R.P., Abbott W.M. (1990). Aberrant right subclavian artery: varied presentations and management options. J Vasc Surg.

[10] Choi Y., Chung S.B., Kim M.S. (2019). Prevalence and anatomy of aberrant right subclavian artery evaluated by computed tomographic angiography at a single institution in korea. J Korean Neurosurg Soc.

[11] Wu Y., Zhang H., Tang C. (2021). Coexistence of an aberrant right subclavian artery and anomalous origins of bilateral vertebral arteries: A case report. Medicine (Baltimore).

[12] Tsai I.C., Tzeng W.S., Lee T., Jan S.L., Fu Y.C., Chen M.C. (2007). Vertebral and carotid artery anomalies in patients with aberrant right subclavian arteries. Pediatr Radiol.

[13] Wang P., Wang Q., Bai C., Zhou P. (2020). Iatrogenic aortic dissection following transradial coronary angiography in a patient with an aberrant right subclavian artery. J Int Med Res.

[14] Dion J.E., Gates P.C., Fox A.J., Barnett H.J., Blom R.J. (1987). Clinical events following neuroangiography: a prospective study. Stroke.

